# VGF Protein and Its C-Terminal Derived Peptides in Amyotrophic Lateral Sclerosis: Human and Animal Model Studies

**DOI:** 10.1371/journal.pone.0164689

**Published:** 2016-10-13

**Authors:** Carla Brancia, Barbara Noli, Marina Boido, Andrea Boi, Roberta Puddu, Giuseppe Borghero, Francesco Marrosu, Paolo Bongioanni, Sandro Orrù, Barbara Manconi, Filomena D’Amato, Irene Messana, Federica Vincenzoni, Alessandro Vercelli, Gian-Luca Ferri, Cristina Cocco

**Affiliations:** 1 NEF-Laboratory, Department of Biomedical Sciences, University of Cagliari, Cagliari, Italy; 2 Neuroscience Institute Cavalieri Ottolenghi, Department of Neuroscience, University of Turin, Turin, Italy; 3 Department of Neurology, Azienda Universitario Ospedaliera di Cagliari and University of Cagliari, Cagliari, Italy; 4 Neurorehabilitation Unit, Neuroscience Department, University of Pisa, Pisa, Italy; 5 Medical Genetics, Department of Medical Sciences, University of Cagliari, Binaghi Hospital, Cagliari, Italy; 6 Department of Life and Environmental Sciences, University of Cagliari, Cagliari, Italy; 7 Institute of Biochemistry and Clinical Biochemistry, Catholic University, Rome, Italy; 8 Institute of Chemistry of the Molecular Recognition CNR, Rome, Italy; Julius-Maximilians-Universitat Wurzburg, GERMANY

## Abstract

VGF mRNA is widely expressed in areas of the nervous system known to degenerate in Amyotrophic Lateral Sclerosis (ALS), including cerebral cortex, brainstem and spinal cord. Despite certain VGF alterations are reported in animal models, little information is available with respect to the ALS patients. We addressed VGF peptide changes in fibroblast cell cultures and in plasma obtained from ALS patients, in parallel with spinal cord and plasma samples from the G93A-SOD1 mouse model. Antisera specific for the C-terminal end of the human and mouse VGF proteins, respectively, were used in immunohistochemistry and enzyme-linked immunosorbent assay (ELISA), while gel chromatography and HPLC/ESI-MS/MS were used to identify the VGF peptides present. Immunoreactive VGF C-terminus peptides were reduced in both fibroblast and plasma samples from ALS patients in an advanced stage of the disease. In the G93A-SOD1 mice, the same VGF peptides were also decreased in plasma in the late-symptomatic stage, while showing an earlier down-regulation in the spinal cord. In immunohistochemistry, a large number of gray matter structures were VGF C-terminus immunoreactive in control mice (including nerve terminals, axons and a few perikarya identified as motoneurons), with a striking reduction already in the pre-symptomatic stage. Through gel chromatography and spectrometry analysis, we identified one form likely to be the VGF precursor as well as peptides containing the NAPP- sequence in all tissues studied, while in the mice and fibroblasts, we revealed also AQEE- and TLQP- peptides. Taken together, selective VGF fragment depletion may participate in disease onset and/or progression of ALS.

## Introduction

Amyotrophic Lateral Sclerosis (ALS) is a progressive and fatal neurodegenerative disorder characterized by selective degeneration and death of upper and lower motor neurons, respectively, in the cerebral cortex, brainstem and spinal cord. *Vgf* is a neutrophin induced gene that encodes for a single VGF precursor, composed of 617 (rat/mouse) / 615 (human) amino acids [1]. Studies in rats revealed that VGF mRNA is widely expressed in areas that are known to degenerate in ALS, including cerebral cortex, spinal cord, and cranial nerve motor nuclei as trigeminal and hypoglossal nuclei [2]. While the role of VGF in the nervous system is yet to be clarified, in VGF knock-out mice, synaptic plasticity and memory would be affected, in addition to a depressive behaviour [3]. VGF cleavage can gives rise to a variety of bioactive peptides, of which those derived from its C-terminal portion have so far been more extensively studied. Two peptides from such region, named AQEE-30 and TLQP-62 appear to regulate synaptic function [4], while TLQP-62 can also induce neurogenesis [5], or enhance neuronal hippocampal transmission [6], and would be required for hippocampal memory consolidation [7]. In the most commonly used murine model of ALS, i.e G93A-SOD1 transgenic mice overexpressing the mutated human SOD1 gene, VGF immunoreactivity was reported to be reduced in the cerebro-spinal fluid (CSF) and serum, as in the spinal cord in parallel with the progression of muscle weakness [8]. In CSF from ALS patients, a VGF-derived 4.8 kDa fragment significantly decreased compared to controls [9], while immunoreactivity of the VGF full-length was reduced in parallel with development of ALS symptoms [8]. Moreover, the density of VGF immunoreactivity was also lower in spinal cords from sporadic ALS patients than in control subjects [10]. Interestingly, increased VGF expression would attenuate excitotoxic injury in primary mixed spinal cord cultures from G93A-SOD1 mice [8]. VGF could be also involved in neuroprotective mechanisms in stress-induced cell death *in vitro* as well as in *vivo* [10]. Recent literature data showed as human primary fibroblast cultures from ALS patients reflect some pathophysiological features observed in neuronal cells [11], with altered bioenergetic properties in neurodegenerative diseases, including ALS [12, 13]. Hence, we decided to use both patients’ plasma and fibroblasts to search for evidence of VGF changes. In order to confirm and extend our study, we also analysed the G93A-SOD1 animal model (spinal cord and plasma). Highly characterized VGF antisera raised against the human and mouse C-terminal end of the VGF were used in immunohistochemistry (IHC) and enzyme-linked immunosorbent assay (ELISA), while gel chromatography and HPLC high-resolution Electron Spray Ionization-MS (HPLC-ESI-MS) and HPLC/ESI-MS/MS (MS/MS) were carried out to identify VGF peptides present.

## Materials and Methods

### VGF Antibodies

We produced two different policlonal VGF antisera against the following human and rat/mouse proVGF C-terminal sequences: (I) human VGF_607-615_: -I EHVL LRRP [14] and (II) rat/mouse VGF_609-617_: -I EHVL LHRP [15]. The relevant rat and human nonapeptide antigens were synthesized by Affiniti-Biomol, Exeter, Devon, UK and conjugated to bovine thyroglobulin *via* an additional N-terminal D-tyrosine for immunizations. Characterization of both antibodies has been previously described in detail [16–19].

### Human plasma

The plasma samples included either female (n = 18) or male (n = 23) ALS patients and age-matched control subjects (n = 45), ranging 25–85 yrs and collected between 2012–14 at the Department of Neurology, Azienda Universitaria-Ospedaliera of Cagliari and University of Cagliari. All participants provided their written informed consent, and the study was approved by the Ethical Committee of Cagliari AOU (“Azienda Ospedaliera Universitaria”), protocol n. 450/09/C.E. The groups of patients analysed included subjects with TDP-43 mutation (n = 19), G93A-SOD1 (n = 3), expansion in C9ORF72 gene (n = 3) or unidentified aetiology (n = 16).

At the time of blood sampling, the patients’ motor and functional status were assessed by neurologists using the Amyotrophic Lateral Sclerosis Functional Rating Scale Revised (ALSFRS-R [20]). Patients’ scores are reported in supporting information (S1 Dataset).

On the basis of their survival and/or clinical state one year after the above blood sampling, patients were assigned to either of two “outcome groups”: (i) “early stage” (n = 23), alive patients which had not yet required tracheostomy; or (ii) “advanced stage” (n = 18), including patients, which had required tracheostomy, or were deceased.

### Human fibroblasts

Primary fibroblast cell cultures were derived from skin biopsies taken with informed consent from: ALS patients at the advanced phase with (I) heterozygous missense TARBDP-A382T mutation TARBDP-A382T; (n = 2), (II) WT genotype at the TARDBP, FUS or C9orf72 loci (n = 2), all compared with healthy age-matched control subjects (n = 3). The use of fibroblast samples was approved by the local ethics committee. Cells were grown in high-glucose DMEM supplemented with 20% (vol/vol) fetal bovine serum, 1% penicillin/streptomycin (10,000 units penicillin and 10 mg streptomycin per mL in 0,9% NaCl) (all from Sigma Aldrich, ST). For immunocytochemistry, the cells were grown on glass coverslips and fixed after treatment in 4% w/v paraformaldehyde (PFA) for 15 min., permeabilized with cold methanol for 5 min. and 0.2% Triton X-100 in phosphate-buffered saline (PBS, 0.01 mol/l PO_4_, pH 7.4) for 20 min. Cells were then incubated for 4h at room temperature with human VGF C-terminus (1:1000) diluted in PBS containing 30ml/l of normal donkey serum and immunoreactivity was revealed with specific donkey secondary antisera conjugated with Cy3. Nuclei were counterstained with Hoechst 33342 (Sigma Aldrich). Coverslips were mounted with Glycerol/PBS (1:1). Negative controls were routinely performed for each experiment, incubating the samples with non-immune serum and/or with the appropriate secondary antisera. Imaging was carried out using an Olympus BX41 fluorescence microscope. For ELISA and gel chromatography, the cells were extracted with PBS-PIC (protease inhibitor cocktail: P8340, Sigma-Aldrich, Schnelldorf, Germany). Since there was not other evidence of the presence of VGF in fibroblasts, the expression of the VGF gene at the level of mRNA was studied by RT-PCR amplification using both patient and control samples. Briefly: 0.5–1μg of total RNA was reverse transcribed to cDNA using the ThermoScript^™^ RT-PCR System for First-Strand cDNA Synthesis (Invitrogen). Two ul of final reaction were used for amplifying a fragment of 160 bp of the VGF gene. The reaction was carried out in 25 μl final volume using the primer pair VGF77 (GACCTCGACCGTCGCTCC) and VGF241 (GCCGACAATCTGAGGGCTTT). HPRT was used as a housekeeping control gene and amplified using the primer pair HumHPRT.1 (CCCTGGCGTCGTGATTAGTG) and HumHPRT.2 (CGAGCAAGACGTTCAGTCCT). Specific primers for the human VGF gene were designed using primer-Blast (http://www.ncbi.nlm.nih.gov/tools/primer-blast/) and validated by RT-PCR carried out on cDNA from SY5Y Human cell lines.

### Animal Model

The experiments were performed on transgenic male mice B6SJL-TgN(SOD1-G93A)1Gur over-expressing human SOD1, containing the Gly93 to Ala mutation (Jackson Laboratory, Bar Harbor, ME, USA; stock number 002726); these mice have a high transgene copy number. The founders were kindly gifted by M. Bentivoglio and R. Mariotti (University of Verona). The colony was derived from breeding male transgenic mice to naive (B6xSJL/J)F1 females (Janvier SAS, Le Genest-Saint-Isle, France). All experimental procedures on live animals were carried out in accordance with the European Communities Council Directive 86/609/EEC (November 24, 1986) Italian Ministry of Health and University of Turin institutional guidelines on animal welfare (law 116/92 on Care and Protection of living animals undergoing experimental or other scientific procedures; authorization number 17/2010-B, June 30, 2010). Additionally, the Ethical Committee of the University of Turin approved this study. All efforts were made to minimize the number of animals used and their suffering. For the genotyping, a 0.5 cm-long specimen of mouse tail was incubated in 100 μl of lysis buffer (10 mM Tris HCl, 50 mM KCl, 0.01% gelatin, 0.45% IGEPAL, 0.4% Tween-20) and 25 μg of proteinase k at 55°C, overnight, under gentle shaking, in order to extract the DNA. Then, PCR was performed to evaluate the presence of the human transgene superoxide dismutase-1 (hSOD1). As suggested by the supplier, the employed primers were: 5’-CATCAGCCCTAATCCATCTGA-3’ and 5’-CGCGACTAACAATCAAAGTGA-3’ for hSOD1 gene; while 5’-CTAGGCCACAGAATTGAAAGATCT-3’ and 5 GTAGGTGGAAATTCTAG CATCATCC-3’ for mouse interleukin 2 gene, as internal control. To characterize the time course and the progression of ALS symptoms, transgenic (TG) mice were weighed weekly and underwent a battery of behavioral tests, starting from the pre-symptomatic phase: neurological test, rotarod and paw grip endurance (PaGE) tests. All tests have been extensively described in Boido et al. [21]. TG animals were subdivided into the following three groups, according to the stage of motor dysfunction progression and to their postnatal (P) days: (i) pre-symptomatic (around P45), (ii) early-symptomatic (around P90; two repeated deficits for two consecutive times) and (iii) late-symptomatic (around P120; 20% weight decrease and inability in performing tests). Age-matched wild type mice (WT) were used as controls.

### Tissue sampling

For ELISA, at each time point, mice (n = 7 per genotype) were euthanized by cervical dislocation, and cervical spinal cords were dissected. Tissues were extracted with ice-cold PBS, containing PIC and homogenized for 3 min. using a micro sample pestle, hence tubes were heated in a vigorously boiling water bath for 10 min., and centrifuged (3,000 rpm, 15 min.). Supernatants were stored frozen until use (−20°C). From the same mice, blood (approximately 200μl) was drawn under terminal anesthesia by cardiac puncture, collected in a tube containing ethylenediaminetetraacetic acid (EDTA, 178 mg/ml), and rapidly centrifuged (11,000 rpm, 5 min.), hence plasma was stored frozen (-80°C). For IHC, at each time point, mice (n = 3 per genotype) were deeply anaesthetized by gaseous anaesthesia (3% isoflurane vaporized in O_2_/N_2_O 50:50), underwent intracardiac perfusion with 4% PFA, pH 7.4. The whole spinal cords were removed and post-fixed in PFA for 2 h at 4°C. After fixation, cervical segments of spinal cords were included in an embedding medium [22] and cut (at 10μm) using a HM-560 cryomicrotome (Microm; Walldorf, Germany).

### ELISA

Competitive ELISA was performed through VGF assays previously used with human and mouse tissues [18]. Multiwell plates (Nunc, Milan, Italy) were coated with the relevant synthetic peptides and treated with PBS (containing 9% normal serum from the secondary antisera donor species, 20nM aprotinin, and 1mg/ml EDTA) for 2 hours. Primary incubations, with either human or rat/mouse VGF C-terminus antiserum, were carried out in duplicate, including serial standard dilutions in parallel with samples from human (plasma, fibroblasts) or mouse (spinal cord, plasma). Biotinylated secondary antibodies (Jackson, West Grove, PA, USA), streptavidin-peroxidase conjugate (Biospa, Milan, Italy), and tetrametylbenzidine (TMB X-traKem-En-Tec, Taastrup, Denmank) as substrate were used to reveal the positive labelling. Hence, the reaction was stopped with HCL (1mol/L) and the optical density was measured at 450nm using a multilabel plate reader (Chameleon: Hidex, Turku, Finland). Recovery of synthetic peptide/s added to plasma, or to tissue samples at extraction was >85% for all assays used. Each VGF assay was characterized using various synthetic peptides. Inter (CV1) and intra (CV2) assays were 3–4% and 9–11%, respectively. Data were expressed as mean ± SEM throughout.

### Statistical analysis

For ELISA data, statistical analyses were carried out by one-way ANOVA, followed by post hoc multiple comparison tests (Student-Newman-Keul test), or by two-tailed Student’s t-test as appropriate by means of the StatistiXL software. Linear regression analysis was used to estimate the possible correlation between the VGF levels and the ALSFRS-R values. P-value < 0.05 was considered significant.

### Immunohistochemistry

Sections of mouse spinal cord samples were incubated overnight in a humid chamber, with the mouse VGF C-terminus primary antiserum diluted (1:4000) in PBS containing 30ml/l of normal donkey serum, 30 ml/l of normal mouse serum and 0.02g/l NaN_3_. Double immunofluorescence experiments were carried out mixing the anti VGF antiserum with the anti rat/mouse VAChT antibody (vesicular acetylcholine transporter; BIOMOL Research lab, Plymouth Meeting, PA; 1:400 dilution), raised in different donor species (rabbit and goat, respectively). The relevant species-specific donkey secondary antisera, conjugated with either Alexa-488 (emitting in green; 1:200) and or cyanin 3.18 (yellow/red; 1:300) (Jackson Immunoresearch Laboratories, West Grove, PA) were used to reveal immunoreactivity of the primary antisera. Slides were covered with PBS-glycerol (40%), observed and photographed using BX41 and BX51 fluorescence microscopes (Olympus, Milan, Italy) equipped with the Fuji S2 and S3 Pro digital cameras (Fujifilm, Milan, Italy). Routine controls included substitution of each antiserum, in turn, with PBS, the use of pre-immune or non-immune sera, and the testing of each secondary antiserum with the respective non-relevant primary antibody.

### Gel Chromatography, HPLC high-resolution ESI-MS and MS/MS analysis

To reveal the MW of the VGF fragments present in the tissues studied, we used a gel chromatography approach that can easily isolate high MW forms (up to 70kDa), including the VGF precursor, but also smaller VGF fragments. In order to validate the findings, we also used HPLC high-resolution ESI-MS to reveal the specific VGF peptide sequences, at least in the mouse spinal cord. For gel chromatography analysis, cervical spinal cord extracts (1.6 mL) and plasma (1mL), both pooled from control mice, as well as plasma (3 mL), and fibroblasts (1.5mL) from control subjects, were individually loaded onto a Sephadex G-50S column (Sigma; 2cm^2^ x 1m). This column was equilibrated with 50 mM ammonium bicarbonate and eluted with the same buffer. A MW marker kit (MWGF70, Sigma) was used for the column calibration. The collected fractions (3 mL) were reduced in volume with a vacufuge concentrator (Eppendorf, Milan, Italy) and assessed by ELISA. The overall recovery of loaded immunoreactivity ranged between 80% and 100%. For HPLC high-resolution ESI-MS and MS/MS analysis, the cervical spinal cord extracts from control mice were fractionated by using 10kDa cutoff Amicon Ultra devices (Merck Millipore, Tullagreen Carrigtwohill Co. Cork, Ireland). Fractions<10kDa were dried in a Vacufuge Concentrator (Eppendorf, Milan, Italy), and a total of 5 μg was resuspended in 50 μl of aqueous formic acid solution (0.1% v/v) and 1 μg analyzed using an Ultimate 3000 RSLCnano coupled with an Orbitrap Elite mass spectrometer (ThermoFisher, San Jose, CA). Separation experiments were performed using an EASY-Spray Column PepMap^®^ RSLC C18 (3 μm particle diameter; column dimension 75 μm ID x 15 cm) with the following eluents: (A) 0.1% (v/v) aqueous formic acid and (B) acetonitrile with 0.1% (v/v) aqueous formic acid. The applied gradient was linear from 0 to 55% of solvent B in 35 min, at a flow rate of 300 nL/min. The Elite-Orbitrap mass spectrometer operated in a data-dependent mode in which each full MS scan (60 000 resolving power) was followed by five MS/MS scans where the first five multiple-charged ions were dynamically selected and fragmented by collision-induced dissociation (CID) at a normalized collision energy of 35%. Tandem mass spectra were analysed using the Thermo Proteome Discoverer 1.4 software, and the SEQUEST cluster (University of Washington, Seattle, WA, licensed by Thermo Electron Corp) was used as search engine against UniProtKB mouse proteome (releases 2016–03). For peptide matching the following limits were used: Xcorr scores greater than 1.5 for singly charged peptide ions and 2.0 and 2.5 for doubly and triply charged ions, respectively. Precursor mass search tolerance was set to 10 ppm, and fragment mass tolerance was set to 0.02 Da. N-Terminal Modification (Gln → pyro-Glu) and C-Terminal Modification (Amidated) were chosen as dynamic modifications. A false discovery rate (FDR) below 1% was applied. Peptide sequences and sites of covalent modifications were also validated by manual spectra annotation performed by comparing experimental MS/MS spectra with the theoretical generated by the MS-Product program available on Protein Prospector website (http://us.expasy.org/tools). The match was considered positive when all the experimental m/z values with a relative abundance higher than 5% were present in the theoretical fragmentation spectrum, and when differences between the experimental and theoretical values were less than ± 0.03 m/z.

## Results

### VGF changes in human

**Fig 1:**
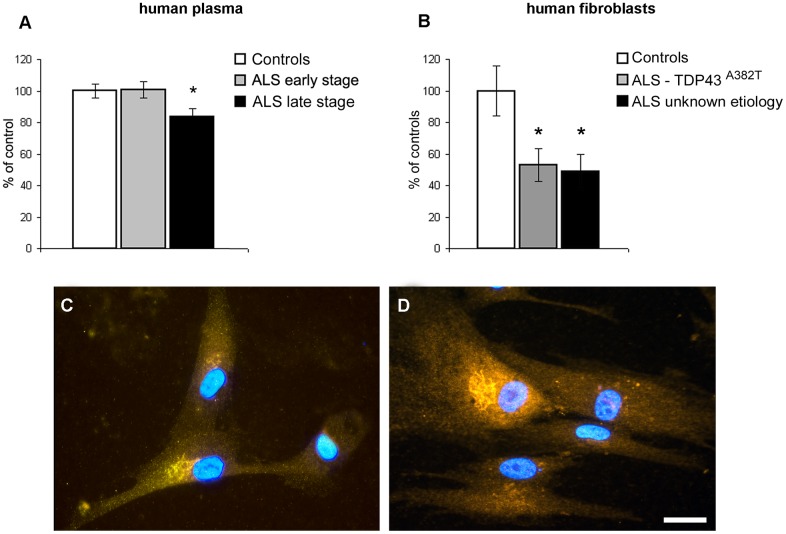
VGF C-terminus peptide changes in human. In plasma (A), ALS patients reveal a reduction in the VGF C-terminus peptide levels seen in the late stage (n = 18) compared to the controls (n = 45) and patients at the early stage (n = 23) (mean ± SEM; percentage of decrease: 16%, p<0.04). In human fibroblasts (B) taken from patients at the late stage of disease, VGF C-terminus peptide levels are significant decreased irrespective to the mutation (mean ± SEM; percentage of decrease 47% p<0.04; 51%, p<0.02 for TDP-43 and unknown etiology, respectively). Values (from 3 sets of experiments for each tissue) are expressed as percent of control level. Immunocitochemistry (C, D) shows the localization of VGF C-terminus peptides into the cytoplasm and especially within the vesicular membrane system in both controls (C) and ALS patients (D). No changes in the VGF immunolocalization were seen between ALS patients and controls. VGF: yellow/red (Cy3), hoechst 33342 (blue): nucleus. Data referring to 4 sets of experiments. Scale bar: 10μm.

Using plasma samples (Fig 1A), we found that VGF C-terminus peptide levels were decreased in ALS patients at the advanced stage (percentage decrease: 16%, p<0.04) compared to controls and patients at the early stage. We also found a VGF C-terminus reduction in fibroblasts (Fig 1B) obtained from ALS patients at the advanced stage, with either TDP-43 mutation or unidentified aetiology (percentage decrease: 47% p<0.04; 51%, p<0.02, respectively), compared to the control cells Raw data from ELISA are reported in S2 Dataset. Instead, the mRNA of the VGF gene was revealed in cells from both patients and controls (not shown). However, due to the very low levels of transcription, all attempts to quantify changes in mRNA levels by Real-Time PCR, have not produced consistent results. Immunocytochemistry (Fig 1C and 1D) revealed the VGF localization into the cytoplasm, close to the nucleus and probably identified as Golgi area, with no differences between control (Fig 1C) and ALS patient (Fig 1D) derived cells.

### Molecular characterization in human

**Fig 2:**
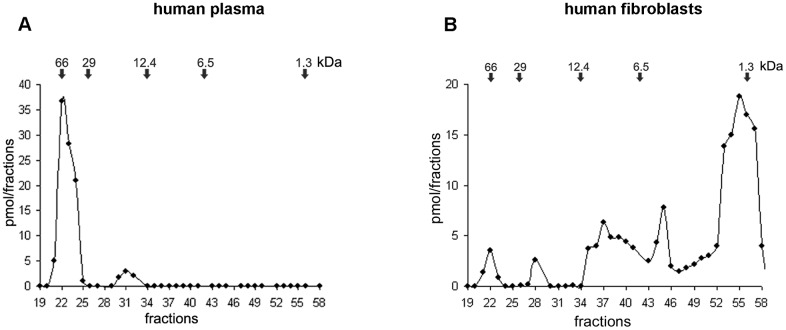
Molecular characterization in human. ELISA coupled with gel chromatography reveals two MW forms of approximately 66 and 14–15 kDa, recognised by the VGF antibody in both plasma (A) and fibroblasts (B), where additional MW forms of 8 kDa, 3–4 kDa, and 1.3–2kDa were also revealed. Result data referred to 3 sets of experiments, using control animal only.

Molecular characterization by ELISA coupled with gel chromatography showed that in both plasma and fibroblasts (Fig 2A and 2B) VGF C-terminus antiserum recognized two fragments, one of 66kDa likely to be the VGF precursor or a high MW VGF protein, and an additional form of 14–15 kDa encompassing a peptide containing the sequence from NAPP up to the C-terminus, made of 129 aa, hence namely NAPP-129. Moreover, in human fibroblasts we revealed also additional forms of: (a) 8kDa corresponding to the TLQP-62, (b) 3–4 kDa, corresponding to the AQEE-30 and (c) one uncharacterized form of 1.3 kDa. It has to be noted that the name of the VGF peptides is expressed here using the single-letter codes of the first four amino acids (at the peptide N-terminus), followed by the number of amino acid residues. The MW forms revealed in human tissues are summarized in Table 1.

**Table 1:** VGF C-terminus fragments found by gel chromatography in human.

Tissue	VGF fragment	Sequence	MW (kDa)
Plasma	VGF protein	VGF_1-615_*	66
	NAPP-129	VGF_485-615_	14–15
Fibroblasts	VGF protein	VGF_1-615_*	66
	NAPP-129	VGF_485-615_	14–15
	TLQP-62	VGF_554-615_	8
	AQEE-30	VGF_586-615_	3–4

MW: molecular weight;

*VGF precursor or a high MW VGF form

### VGF levels and ALS Functional Rating Scale

We analysed patients in order to correlate the VGF C-terminus levels with the ALSFRS-R values. We observed a no statistically significant linear relationship (p = 0.58, r = 0.086) between VGF levels and ALSFRS-R values (S1 Fig). At the time of blood sampling, the two groups had not significant differences in their ALSFRS-R values (p = 0.091).

### VGF changes in mouse

**Fig 3:**
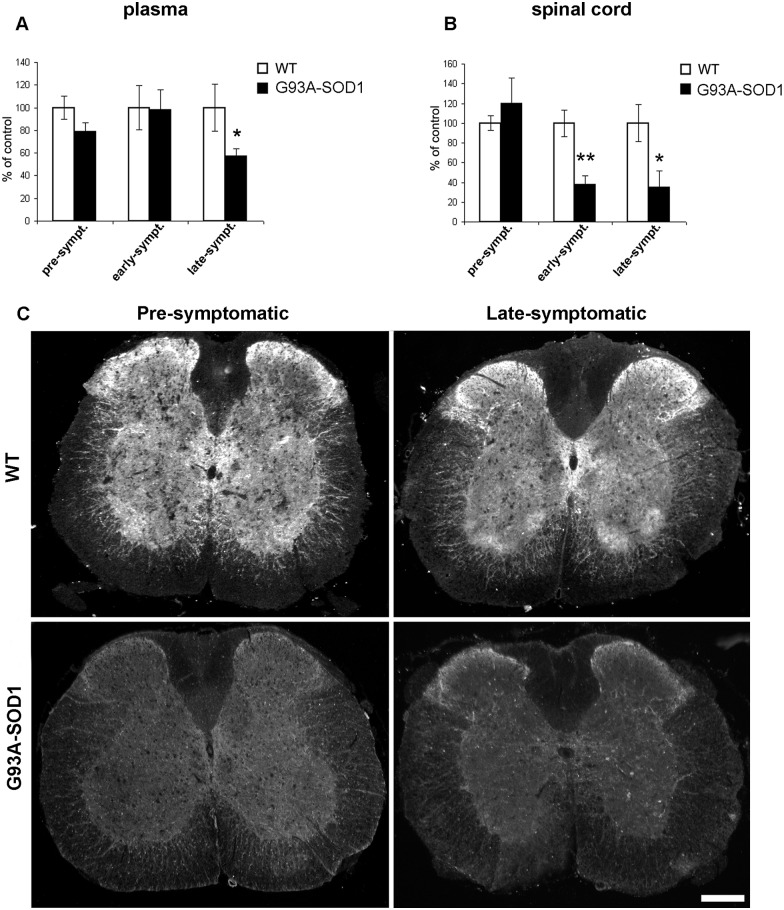
VGF C-terminus peptide changes in mouse. In plasma (A), the G93A-SOD1 mice show a VGF reduction revealed in the late symptomatic phase compared to the WT mice (43% reduction, p<0.01, data from 3 sets of experiments), while, in the spinal cord (B), a loss of immunoreactivity is shown since in the early- other than in the late-symptomatic phases of G93A-SOD1 mice compared to the corresponding WT (**p<0.004 and *p<0.03, respectively; from 3 sets of experiments). In immunohistochemistry, (C) the WT mice (at both pre- and late-symptomatic stages, n = 3 animals per each group), reveal a bright VGF C-terminus immunoreactivity, widely distributed in a high number of nerve structures (terminals, axons, dendrites) within the entire gray matter, including the ventral and dorsal horns and near the III ventricle. Instead, VGF immunostaining disappears in almost all nerve structures in the G93A-SOD1 mice, already in the pre-symptomatic stage (result data from 4 sets of experiments for each case). Scale bars: 100 μm.

**Fig 4:**
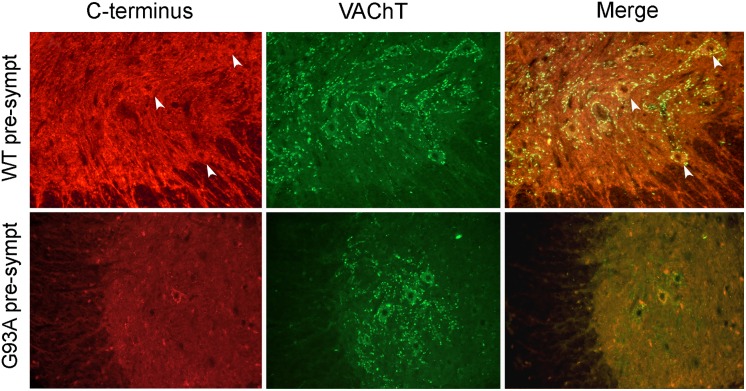
Phenotype of the VGF immunoreactive perikarya. Immunohistochemistry in the ventral horn (A), reveals that in the WT mice, at the pre- symptomatic stage, the VGF antibody labels a number of cell bodies other than axons and terminals. The VAChT instead stains exclusively perikarya in a large number and with a punctate labeling around the cell membranes. All perikarya positive for the VGF antibody were identified as motor neurons (merge panel) by the VAChT labelling (arrows identify the cell bodies positive for both antibodies). Instead, in the G93A-SOD1 mice, at the same pre-symptomatic stage, the VGF staining is strikingly reduced also in motor neurons while the VAChT staining instead, remains visible. The cell body feebly reactive for VGF is identified as motor neuron through a double staining. Red (Cy3) and green (Alexa-488) staining: VGF C-terminus and VAChT, respectively. Result data are from 4 sets of experiments for each case. Scale bars: 50 μm.

In mouse plasma (Fig 3A), we found a VGF C-terminus immunoreactivity reduction in the late-symptomatic phase of the G93A-SOD1 mice, (percentage decrease: 43%, p<0.01), compared to their normal littermates. In the spinal cord instead (Fig 3B), VGF C-terminus immunoreactivity was significantly decreased already in the early-symptomatic phase (62% reduction, **p<0.004,), and remained down-regulated in the late-symptomatic phase (65% reduction, *p<0.03,) of the G93A-SOD1 mice as compared to their normal littermates. Raw data from ELISA are reported in S2 Dataset. In immunohistochemistry (Fig 3C), at either pre- or late- symptomatic stage of the WT mice, VGF C-terminus antiserum labelled a large number of nerve terminals and axons through the entire grey matter including the ventral and dorsal horns. Few perikarya were also labelled within the lamina X surrounding the central canal, as well as within the ventral horns. In the G93A-SOD1 mice instead, the VGF C-terminus immunoreactivity was reduced already in the pre-symptomatic stage. The lack of the VGF immunoreactivity was observed within the entire grey matter, including ventral and dorsal horns, and was equally slight also in the late-symptomatic stage. In order to identify the motor neuronal phenotype of the VGF reactive perikarya in the ventral horns, we also carried out double staining mixing the VGF C-terminus and VAChT antibodies (Fig 4). In the WT mice, almost all the cell bodies positive for the VGF antiserum in the ventral horns were identified as motor neurons. As expected, the general scarcity of VGF immunoreactivity in the pre-symptomatic stage of the G93A-SOD1 mice, included the motor neurons that were reduced in number and/or brightness while the VAChT labelling remained visible.

### Molecular characterization in mouse

**Fig 5:**
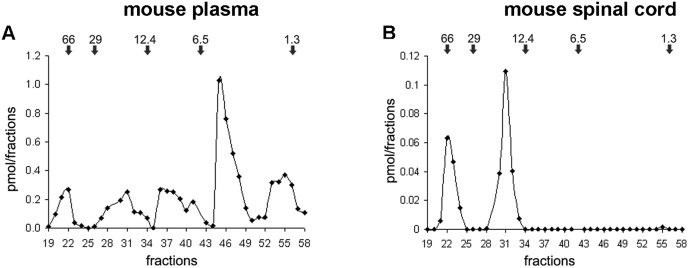
Molecular characterization in mouse. Gel chromatography coupled with ELISA reveals two MW forms of 66 and 14–15 kDa revealed in both plasma (A) and spinal cord (B) plus the following forms of 8, 3–4, and 1.3–2 kDa, visible in plasma only. Result data are from 3 sets of experiments per each tissue, using control animal only. Values are expressed as percent of control level (mean ± SEM).

Gel chromatography coupled with ELISA, in plasma (Fig 5A) and spinal cord (Fig 5B) revealed the same two forms of 66 and 14–15 kDa corresponding to proVGF and NAPP-129, seen in human tissues (both plasma and fibloblasts). Moreover, exclusively in the plasma, we also revealed the same forms seen in fibroblasts and corresponding to TLQP-62, and AQEE-30 as well as one uncharacterized form of 1.5–2 kDa. As mentioned (see materials and methods), to reveal VGF peptide sequences in the mouse spinal cord, we also used the HPLC high-resolution ESI-MS in order to validate the gel chromatography findings. Hence, using HPLC-ESI-MS we revealed short peptides, including two of 1.54 and 2.5kDa, namely AQEE-13, and NAPP-19, respectively, largely encompassing the sequences revealed by gel chromatography, but truncated at their C-terminus, and, a novel peptide of 2.5kDa that we called ELQE-20. The MW forms revealed in mouse tissues are summarized in Table 2. The results of HPLC-ESI-MS/MS experiments, and annotated spectra are reported in S3 Dataset.

**Table 2:** VGF C-terminus fragments found in mouse.

Tissue	VGF fragment	Sequence	MW (kDa)	Method
Plasma	VGF protein	VGF_1-617_*	66	SEC + ELISA
	NAPP-129	VGF_489-617_	14–15	SEC + ELISA
	TLQP-62	VGF_556-617_	8	SEC + ELISA
	AQEE-30	VGF_588-617_	3–4	SEC + ELISA
Spinal cord	VGF protein	VGF_1-617_*	66	SEC + ELISA
	NAPP-129	VGF_489-617_	14–15	SEC + ELISA
	NAPP-19	VGF_489-507_	1.9	HPLC-ESI-MS
	AQEE-13	VGF_588-600_	1.54	HPLC-ESI-MS
	ELQE-20	VGF_353-372_	2.5	HPLC-ESI-MS

MW: molecular weight; SEC: Size-exclusion chromatography, HPLC-ESI-MS:RP-HPLC high-resolution ESI-MS and MS/MS analysis

* VGF precursor or a high MW VGF form

## Discussion

We report here the first evidence of a VGF down-regulation in both plasma and fibroblasts from ALS patients, occurred at the advanced disease phase. These findings are supported by the decrease in the VGF levels showed also in plasma from the ALS mouse model. However, in mice, reduced plasma VGF levels during the late phase were preceded by an early VGF down-regulation in the spinal cord.

### VGF in ALS patients

We describe here the first evidence of VGF changes in plasma and fibroblasts from ALS patients. Indeed, the previous studies were so far carried out exclusively using CSF, reporting changes related to either the VGF full length (hence containing the C-terminus portion) [8], or one VGF fragment of 4.8 kDa not containing the C-terminus portion [9]. However, while in our study, the plasma VGF content decreased at a late phase of the disease, the above mentioned studies using CSF, reported a reduction seen at the early stage. Hence, it seems that the VGF reduction occurs first in the CSF and afterward in the plasma. Hence, since the VGF levels were decreased in the advanced group without a statistically significant linear relationship with the functional rating scale, it seems that VGF could be associated with the ensuing progression, rather than to the severity of the disease at the time of blood sampling. Frequently occurs that one candidate biomarker is related exclusively to certain clinical aspects. For instance, the plasmatic Cystatin C levels, were found useful as an indicator of the disease severity and the site of symptom onset [23]. However, they were significantly changed not exclusively in ALS, but also in other neurodegenerative diseases, failing to be identified as a diagnostic biomarker specific for ALS [24]. At this point, one could speculate that, the combination of the VGF with a panel of different biomarkers [25] perhaps including Cystatin, may constitute a better strategy to aid the diagnosis and prognosis of ALS but also its severity classification, as well as predict its progression. However, the exact mechanisms behind the VGF correlation with the disease progression have not been conclusive, however the possible use of VGF as a prognostic factor should be better elucidated. Further studies will be warranted, including the analysis of the other neurodegenerative diseases to confirm the peculiarity of our changes in ALS.

The VGF changes in fibroblasts from ALS patients represent an intriguing and novel finding, since fibroblasts from ALS patients reflect some pathophysiological features observed in neuronal cells [11]. This is the first evidence of the presence of VGF mRNA, as well as VGF protein and its peptides in the fibroblasts. Interestingly, changes in bioenergetic properties as well as mitochondrial morphology and functionality have been found in fibroblasts from ALS patients [26, 27]. Since VGF ablation affects mitochondrial morphology, and mitochondrial number, at least in BAT [28], the VGF reduction in ALS fibroblasts could represent the starting point for further studies in order to investigate the role of VGF in ALS-related mitochondria mechanisms.

### VGF in ALS mice

The VGF plasma reduction seen in mouse was similarly observed in human, however, our result is not according with the previous study that revealed an “early” VGF decrease in the serum from the same G93A-SOD1 mice [8]. These contrasting results are probably due to the different antibodies used through, an antiserum against the AQEE-30 [8] *versus* our antiserum raised against the nonapeptide encompassing the C-terminus portion. However, MW characterization was not carried out in that study [8]. Instead, our VGF antibody recognised AQEE peptides but also the putative proVGF, plus other VGF peptides largely containing the C-terminus portion. In the spinal cord of the G93A-SOD1, using IHC, we revealed the presence of the VGF C-terminus immunoreactivity within the entire gray matter. This represents a novel finding since in the previous study [8] the immunolocalization using an antibody against the VGF N-terminus, was restricted to motor neurons. The hallmark of ALS is the selective damage of the spinal cord motor neurons. however, sensory neuropathy, abnormalities in somatosensory evoked potentials, reduced spinal cord conduction velocity have also been detected in up to 23% ALS patients [29–31] suggesting that ALS may be a multi-system neurodegenerative disease. Furthermore, sensory impairment has been also reported in the SOD1-G93A mice relatively early in disease progression [32]. Interestingly, in the dorsal horn, prolonged up-regulation of VGF mRNA and VGF protein was observed in the rat spared injury model of neuropathic pain [33]. It is conceivable that the early down-regulation of VGF protein in the spinal cord may be related not only to the motor neuron degeneration, but also to other altered spinal cord connections, including sensory pathways.

### Identification of VGF protein and C-terminal peptides

In all tissues tested, we revealed the presence of a 66 kDa-VGF large protein, may compatible to the VGF precursor as well as a MW fraction of 14–15kDa may compatible with the C-terminus peptide that we called NAPP-129 (from the NAPP sequence up to the C-terminus). However, to ensure our results, we also used mass spectrometry in the spinal cord, keeping in mind that it is unable to detect the sequences of large molecules as the VGF precursor (66Kda). In the spinal cord, the mass spectrometry, revealed the presence of the NAPP-19. The NAPP-19 [18], corresponds to the first 19 N-terminal amino acids of the larger size peptide, hypothesized to be NAPP-129 by gel chromatography. Hence the mass spectrometry, as expected, failed to reveal the VGF precursor, but ensures the gel chromatography results, at least in the spinal cord. Moreover, the mass spectrometry, revealed also two further peptides: the AQEE-13 a short peptide overlapping the N-terminus region of the AQEE-30 revealed by gel chromatography in the mouse plasma, while the ELQE-20 is a VGF peptide recently identified in neuroblastoma cells [34]. By gel chromatography we also revealed further peptides, the TLQP-62 and AQEE-30, exclusively in fibroblasts and mouse plasma, suggesting a possible difference in tissue-specific VGF processing. Besides, all the VGF peptides recognised by gel chromatography, could be potentially involved in the changes that we revealed, hence hypothetically related to the ALS pathological mechanisms.

## Conclusions

We reported here a VGF down-regulation in ALS, probably due to the decrease of the large VGF protein of 66kDa, and/or the other smaller C-terminal VGF peptides identified as NAPP-129, TLQP-62, and AQEE-30. As mentioned, AQEE-30 and TLQP-62 peptides revealed bioactivity into the nervous system [4, 5]. In particular, the TLQP-62 enhanced neuronal transmission through a BDNF dependent pathway [6, 7]. The correlation between VGF and BDNF, is also ensured by the abnormalities in the VGF mutant mice that are associated with BDNF deficiency [6], while *vgf* gene is up regulated by BDNF in association with synaptic plasticity [4]. Since neurotrophic factors are crucial for motor neuron survival [35], their decrease is one of the hypothesized causes of the neuronal death in ALS [36]. Hence, it could be speculated that the decrease in the C-terminal VGF peptides may participate in the ALS disease onset and/or progression, through their alteration in BDNF related mechanisms. The understanding of the bioactivity of the C-terminal VGF peptides in the spinal cord may increase the knowledge of the ALS physiopathology mechanisms and may be useful for new therapeutic approaches. Furthermore, since different peptides are produced from the same VGF precursor, the search of a single VGF fragment changing early in the blood and/or in fibroblasts is our future goal, since both tissues, unlike neuronal tissues or CSF, are easily available and may be useful for rapid diagnosis and prognostic purposes.

## Supporting Information

S1 Dataset Patients score in ALSFRS-R. (TIF)

S2 Dataset ELISA in human plasma, fibroblasts, mouse plasma and spinal cord. (TIF)

S3 Dataset Analysis of the extract of control mouse spinal cord by HPLC high-resolution ESI-MS and elaboration of MS/MS data by Proteome Discoverer. -AQEE-13 (AQEEADAEERRLQ; exp. monoisot. m/z 1544.73±0.02 [M+H]+, theor. monoisot. m/z 1544.73, [M+H]+). -NAPP-19 (NAPPEPVPPPRAAPAPTHV; exp. monoisot. m/z 1915.02±0.02 [M+H]+, theor. monoisot. m/z 1915.02, [M+H]+). -ELQE-20 (ELQETQQERENEREEEAEQE; exp. monoisot. m/z 2533.09±0.02 [M+H]+, theor. monoisot. m/z 2533.09, [M+H]+).
(PDF)

S1 Fig VGF levels and ALSFRS-R. (TIF)
