# Supplementary figures and images for: VGF Protein and Its C-Terminal Derived Peptides in Amyotrophic Lateral Sclerosis: Human and Animal Model Studies
Source: PLoS One. 2016 Oct 13;11(10):e0164689. doi: 10.1371/journal.pone.0164689 (PMC5063282; doi:10.1371/journal.pone.0164689)

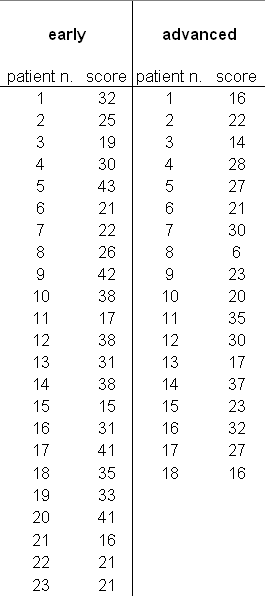

Supplement: S1 Dataset — (TIF) [file pone.0164689.s001.tif]

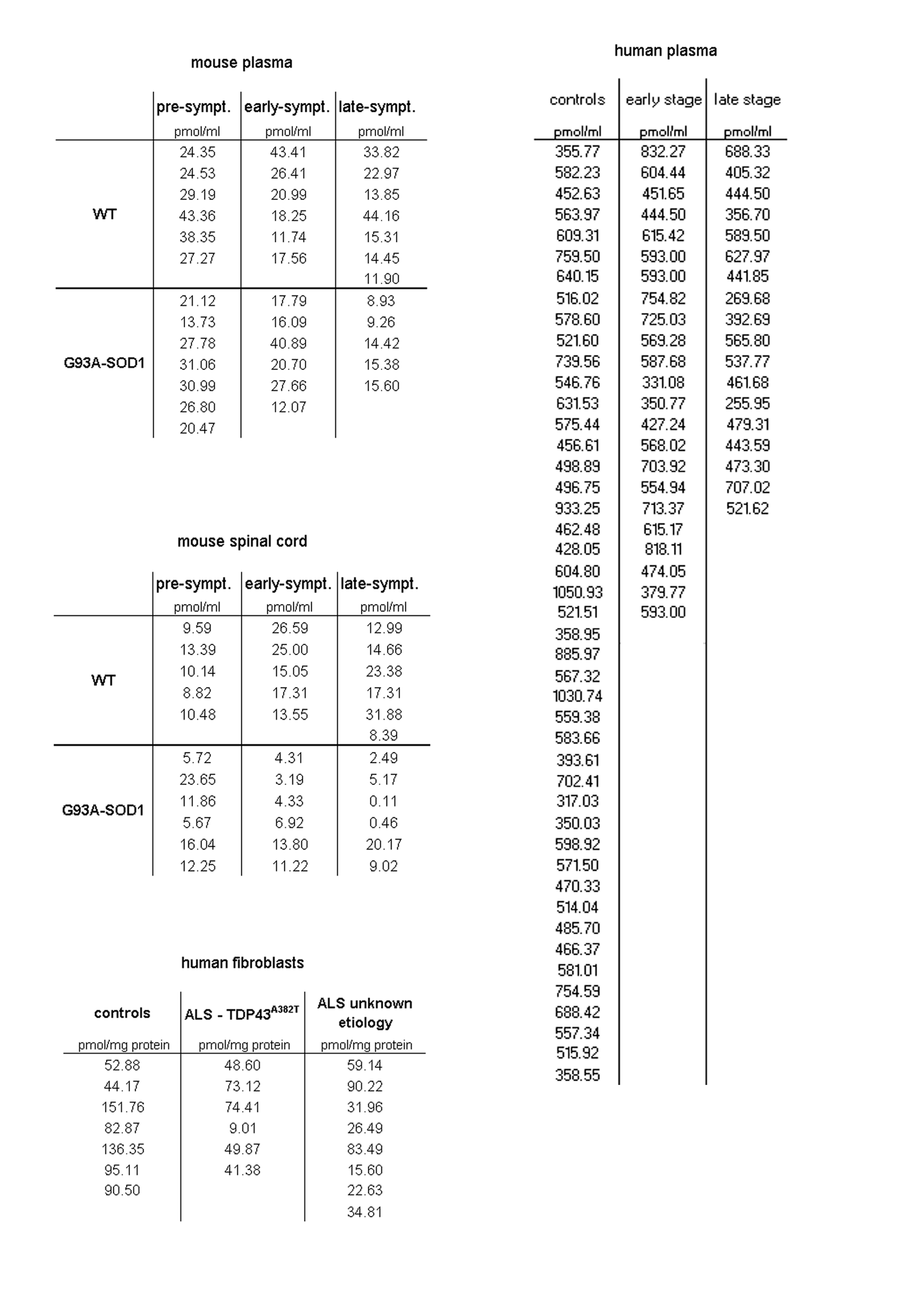

Supplement: S2 Dataset — (TIF) [file pone.0164689.s002.tif]

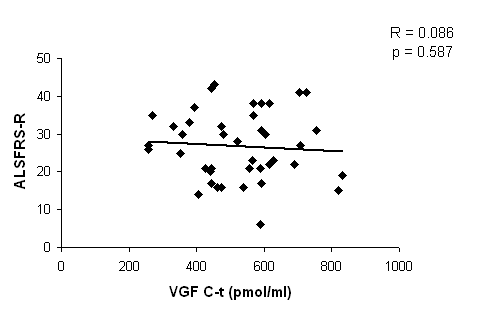

Supplement: S1 Fig — (TIF) [file pone.0164689.s004.tif]
