# Supplementary material for: VGF Protein and Its C-Terminal Derived Peptides in Amyotrophic Lateral Sclerosis: Human and Animal Model Studies
Source: PLoS One. 2016 Oct 13;11(10):e0164689. doi: 10.1371/journal.pone.0164689 (PMC5063282; doi:10.1371/journal.pone.0164689)

AQEE-13

Sequence: **AQEEADAEERRLQ**, Charge: +3, Monoisotopic m/z: 515.58179 Da (+0.26 mmu/+0.51 ppm), MH+: 1544.73081 Da, RT: 15.58 min,
Identified with: Sequest HT (v1.3); XCorr:1.90, Ions matched by search engine: 0/0
Fragment match tolerance used for search: 0.02 Da
Fragments used for search: b; b-H_2O; b-NH_3; y; y-H_2O; y-NH_3

Protein references (1):
- MCG18019 OS=Mus musculus GN=Vgf PE=1 SV=1 - [Q0VGU4_MOUSE]

#1	b^{+}	b^{2+}	b^{3+}	Seq.	y^{+}	y^{2+}	y^{3+}	#2
1	72.04440	36.52584	24.68632	A				13
2	200.10298	100.55513	67.37251	Q	1473.69290	737.35009	491.90248	12
3	329.14558	165.07643	110.38671	E	1345.63432	673.32080	449.21629	11
4	458.18818	229.59773	153.40091	E	1216.59172	608.79950	406.20209	10
5	529.22530	265.11629	177.07995	A	1087.54912	544.27820	363.18789	9
6	644.25225	322.62976	215.42227	D	1016.51200	508.75964	339.50885	8
7	715.28937	358.14832	239.10131	A	901.48505	451.24616	301.16653	7
8	844.33197	422.66962	282.11551	E	830.44793	415.72760	277.48749	6
9	973.37457	487.19092	325.12971	E	701.40533	351.20630	234.47329	5
10	1129.47569	565.24148	377.16341	R	572.36273	286.68500	191.45909	4
11	1285.57681	643.29204	429.19712	R	416.26161	208.63444	139.42539	3
12	1398.66088	699.83408	466.89181	L	260.16049	130.58388	87.39168	2
13				Q	147.07642	74.04185	49.69699	1

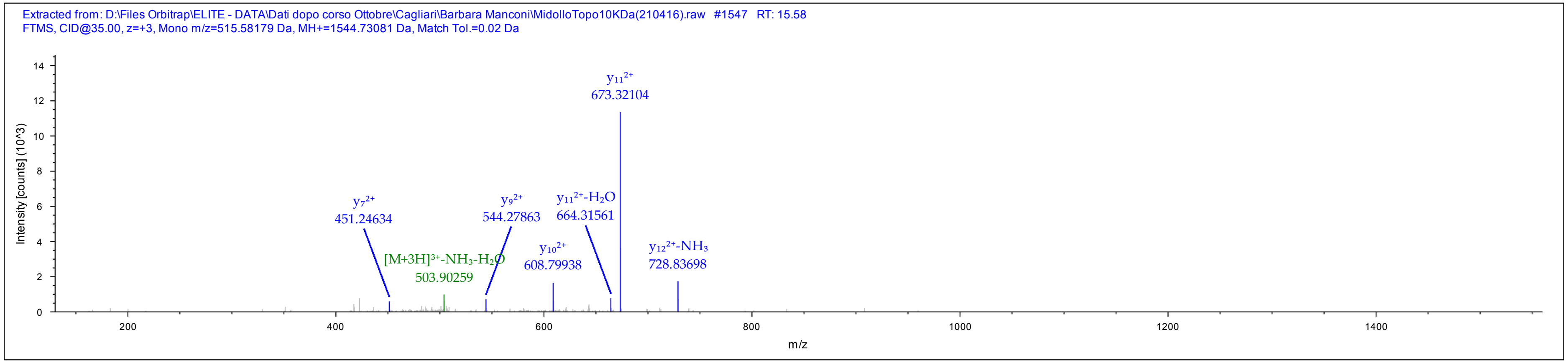

NAPP-19

Manual annotation of Sequence: NAPPEPVPPPRAAPAPTHV, Charge: +3, Monoisotopic m/z: 639.01263 Da (+1.6 mmu/+2.5 ppm), MH+: 1915.02335 Da, RT: 17.94 min,

b	b+2				y	y+2
---	---	1	N	19	---	---
186.0873	---	2	A	18	1800.9755	900.9914
283.1401	---	3	P	17	1729.9384	865.4728
380.1928	---	4	P	16	1632.8857	816.9465
509.2354	---	5	E	15	1535.8329	768.4201
606.2882	---	6	P	14	1406.7903	703.8988
705.3566	---	7	V	13	1309.7375	655.3724
802.4094	---	8	P	12	1210.6691	605.8382
899.4621	---	9	P	11	1113.6164	557.3118
996.5149	---	10	P	10	1016.5636	508.7854
1152.6160	576.8116	11	R	9	919.5108	460.2591
1223.6531	612.3302	12	A	8	763.4097	382.2085
1294.6902	647.8488	13	A	7	692.3726	346.6899
1391.7430	696.3751	14	P	6	621.3355	311.1714
1462.7801	731.8937	15	A	5	524.2827	262.6450
1559.8329	780.4201	16	P	4	453.2456	227.1264
1660.8806	830.9439	17	T	3	356.1928	178.6001
1797.9395	899.4734	18	H	2	255.1452	128.0762
---	---	19	V	1	118.0863	---

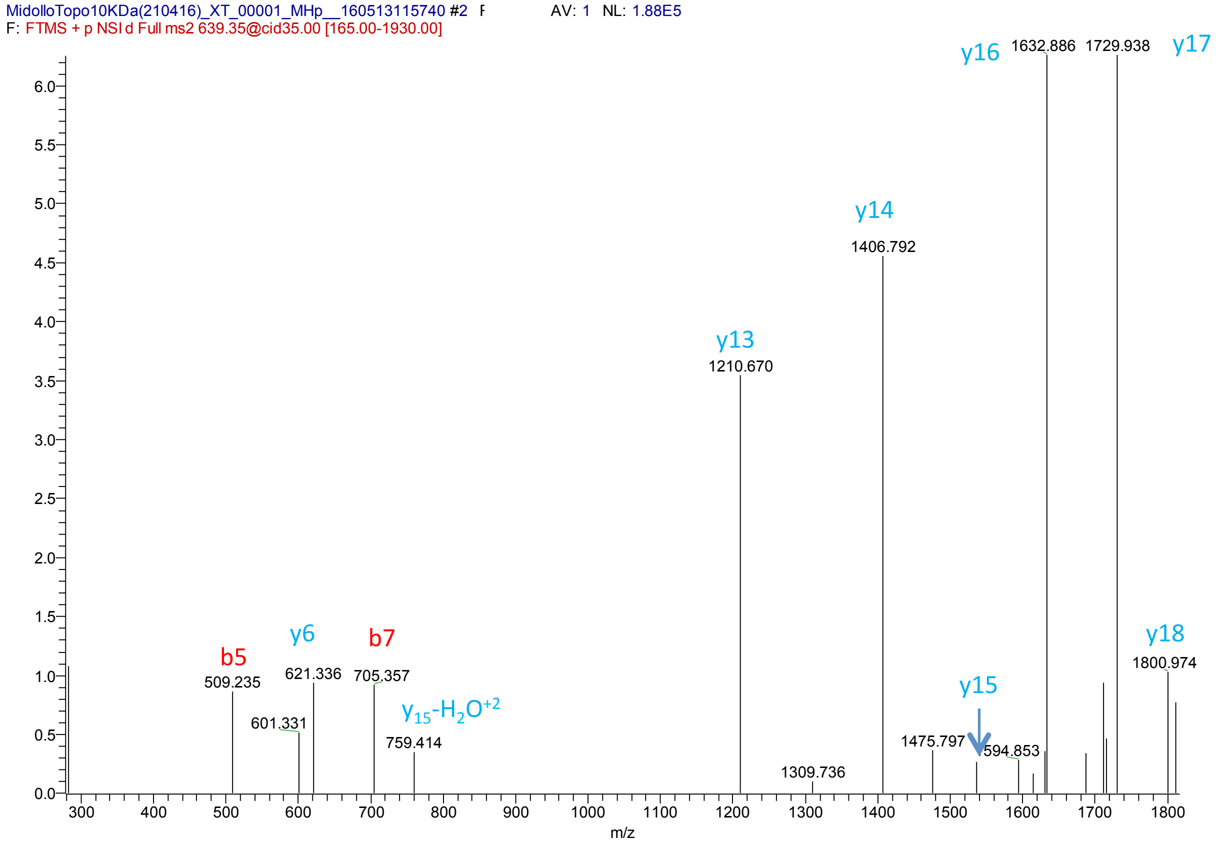

ELQE-16

Sequence: **ELQETQQERENEREEEAEQE**, Charge: +3, Monoisotopic m/z: 845.03760 Da (+2.01 mmu/+2.38 ppm), MH+: 2533.09824 Da, RT: 15.74 min,
Identified with: Sequest HT (v1.3); XCorr:2.72, Ions matched by search engine: 0/0
Fragment match tolerance used for search: 0.02 Da
Fragments used for search: b; b-H_2O; b-NH_3; y; y-H_2O; y-NH_3

Protein references (1):
- MCG18019 OS=Mus musculus GN=Vgf PE=1 SV=1 - [Q0VGU4_MOUSE]

#1	b^+	b^{2+}	b^{3+}	Seq.	y^+	y^{2+}	y^{3+}	#2
1	130.04988	65.52858	44.02148	E				20
2	243.13395	122.07061	81.71617	L	2404.04960	1202.52844	802.02138	19
3	371.19253	186.09990	124.40236	Q	2290.96553	1145.98640	764.32669	18
4	500.23513	250.62120	167.41656	E	2162.90695	1081.95711	721.64050	17
5	601.28281	301.14504	201.09912	T	2033.86435	1017.43581	678.62630	16
6	729.34139	365.17433	243.78531	Q	1932.81667	966.91197	644.94374	15
7	857.39997	429.20362	286.47151	Q	1804.75809	902.88268	602.25755	14
8	986.44257	493.72492	329.48571	E	1676.69951	838.85339	559.57135	13
9	1142.54369	571.77548	381.51941	R	1547.65691	774.33209	516.55715	12
10	1271.58629	636.29678	424.53361	E	1391.55579	696.28153	464.52345	11
11	1385.62922	693.31825	462.54792	N	1262.51319	631.76023	421.50925	10
12	1514.67182	757.83955	505.56212	E	1148.47026	574.73877	383.49494	9
13	1670.77294	835.89011	557.59583	R	1019.42766	510.21747	340.48074	8
14	1799.81554	900.41141	600.61003	E	863.32654	432.16691	288.44703	7
15	1928.85814	964.93271	643.62423	E	734.28394	367.64561	245.43283	6
16	2057.90074	1029.45401	686.63843	E	605.24134	303.12431	202.41863	5
17	2128.93786	1064.97257	710.31747	A	476.19874	238.60301	159.40443	4
18	2257.98046	1129.49387	753.33167	E	405.16162	203.08445	135.72539	3
19	2386.03904	1193.52316	796.01786	Q	276.11902	138.56315	92.71119	2
20				E	148.06044	74.53386	50.02500	1

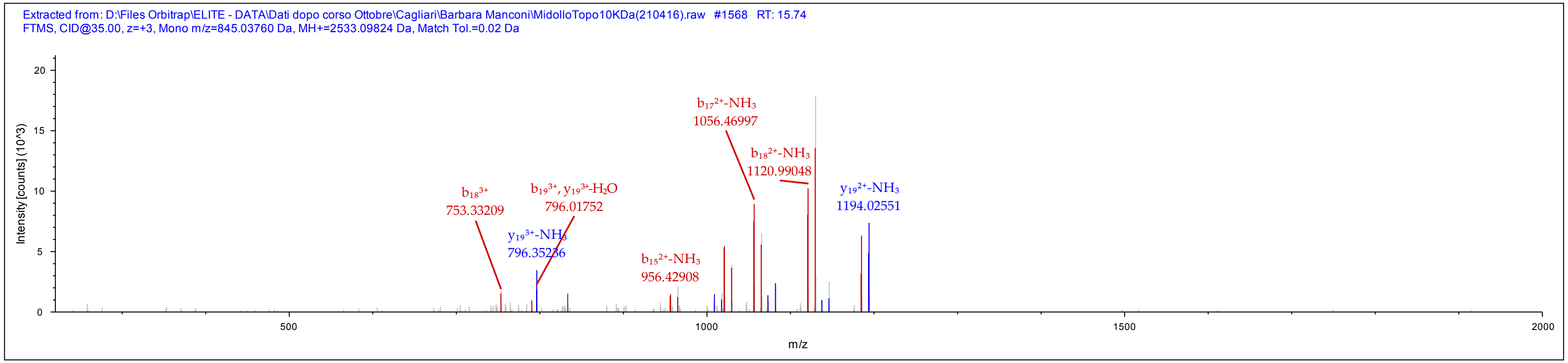

Supplement: S3 Dataset — -AQEE-13 (AQEEADAEERRLQ; exp. monoisot. m/z 1544.73±0.02 [M+H]+, theor. monoisot. m/z 1544.73, [M+H]+). -NAPP-19 (NAPPEPVPPPRAAPAPTHV; exp. monoisot. m/z 1915.02±0.02 [M+H]+, theor. monoisot. m/z 1915.02, [M+H]+). -ELQE-20 (ELQETQQERENEREEEAEQE; exp. monoisot. m/z 2533.09±0.02 [M+H]+, theor. monoisot. m/z 2533.09, [M+H]+). (PDF) [file pone.0164689.s003.pdf]
